# Enhanced tryptophan-kynurenine metabolism via indoleamine 2,3-dioxygenase 1 induction in dermatomyositis

**DOI:** 10.1007/s10067-022-06263-3

**Published:** 2022-07-01

**Authors:** Dan Wu, Mengya Chen, Shile Chen, Shimin Zhang, Yongheng Chen, Qian Zhao, Ke Xue, Feng Xue, Xiaosong Chen, Min Zhou, Hao Li, Jie Zheng, Yunchen Le, Hua Cao

**Affiliations:** 1 Department of Dermatology, Rui Jin Hospital, Shanghai Jiao Tong University School of Medicine, No. 197, Rui Jin 2nd Road, Shanghai, 200025 China; 2 Comprehensive Breast Health Center, Rui Jin Hospital, Shanghai Jiao Tong University School of Medicine, Shanghai, China; 3 Department of Respiratory and Critical Care Medicine, Rui Jin Hospital, Shanghai Jiao Tong University School of Medicine, Shanghai, China; 4 Department of Oncology, Rui Jin Hospital, Shanghai Jiao Tong University School of Medicine, Shanghai, China

**Keywords:** Dermatomyositis, Immune regulation, Indoleamine 2,3-dioxygenase 1, Kynurenine, Tryptophan

## Abstract

**Objectives:**

Extrahepatic tryptophan (Trp)-kynurenine (Kyn) metabolism via indoleamine 2,3-dioxygenase 1 (IDO1) induction was found to be associated with intrinsic immune regulation. However, the Trp-Kyn metabolism–associated immune regulation in dermatomyositis (DM) remains unknown. Therefore, we aimed to investigate the clinical relevance of the Trp-Kyn metabolism via IDO1 induction in DM.

**Methods:**

Liquid chromatography-mass spectrometry (HPLC–MS) was used to examine the serum Kyn and Trp concentrations in DM. In addition, we used X-tile software to determine the optimal cutoff value of the Kyn/Trp ratio, a surrogate marker for Trp-Kyn metabolism. Spearman analysis was performed to evaluate the association of Trp-Kyn metabolism with muscle enzymes and inflammatory markers.

**Results:**

DM patients had significantly higher serum Kyn/Trp ratio (× 10^−3^) when compared with the healthy controls. The serum Kyn/Trp ratio was positively correlated with the levels of muscle enzymes and inflammatory markers. In addition, the serum Kyn/Trp ratio significantly decreased (36.89 (26.00–54.00) vs. 25.00 (18.00–37.00), *P* = 0.0006) after treatment. DM patients with high serum Kyn/Trp ratio had a significantly higher percentage of muscle weakness symptoms (62.5% vs. 20.0%, *P* = 0.019) and higher levels of LDH (316.0 (236.0–467.0) vs*.* 198.0 (144.0–256.0), *P* = 0.004) and AST (56.5 (35.0–92.2) vs. 23.0 (20.0–36.0), *P* = 0.002)) than those with low serum Kyn/Trp ratio. Multiple Cox regression analyses identified ln(Kyn/Trp) (HR 4.874, 95% *CI* 1.105–21.499, *P* = 0.036) as an independent prognostic predictor of mortality in DM.

**Conclusions:**

DM patients with enhanced Trp-Kyn metabolism at disease onset are characterized by more severe disease status and poor prognosis. Intrinsic immune regulation function via enhanced Trp-Kyn metabolism by IDO1 induction may be a potential therapeutic target in DM.

**Table:** 

**Key Points** *• HPLC–MS identified increased serum Kyn/Trp ratio in DM patients, which positively correlated with levels of muscle enzymes and inflammatory markers and was downregulated upon treatment.* *• Cox regression analyses identified ln(Kyn/Trp) as an independent prognostic predictor of mortality in DM.* *• Monitoring intrinsic immune regulation function should be considered a potential therapeutic target in DM patients.*

**Supplementary Information:**

The online version contains supplementary material available at 10.1007/s10067-022-06263-3.

## Introduction

Dermatomyositis (DM) is an autoimmune inflammatory disorder characterized by the presence of pathognomonic skin lesions (heliotrope rash, Gottron’s papules/sign, poikiloderma, etc.) with or without muscular and extra-muscular manifestations (primarily lung involvement, followed by heart, esophagus, gastrointestinal tract, etc.) [1]. Disease activity is reflected by levels of muscle enzymes or inflammatory markers. Moreover, interstitial lung disease (ILD) and malignancy are frequently correlated with disease severity and prognosis [2–4].

Autoimmune-mediated pathological inflammation plays a crucial role in the pathogenesis of DM [5]. Corresponding anti-inflammatory therapeutic strategies, including glucocorticoids and immunosuppressants, were widely used in clinical practice [6, 7]. However, the immune system itself can limit excessive inflammatory responses in normal physiology, termed “immune regulation.” Immune homeostasis is a highly dialectical situation comprising immune regulation and inflammation. They interlace with each other and once the balance is broken, disease occurs. The function of immune regulation would have also altered under the disease conditions, and we propose that the intrinsic immune regulation function should provoke new ideas for disease monitoring and treatment.

The extrahepatic tryptophan (Trp)-kynurenine (Kyn) metabolism is a crucial metabolic foundation underlying the intrinsic immune regulation function [8]. In the later phase of inflammation, IFN-γ induces indoleamine 2,3-dioxygenase 1 (IDO1), the rate-limiting enzyme for Trp-Kyn metabolism, in extensive cells (monocytes, macrophages, plasmacytoid dendritic cells, etc.) converting Trp to Kyn to drive the intrinsic immune regulation function. Impairment in IDO1 induction exacerbates the underlying inflammatory pathology, and reversing this defect would cause improvement [9]. The serum Kyn/Trp ratio equals to IDO1 activity and allows an estimation for level of Trp-Kyn metabolism, essentially serving as an indicator of intrinsic immune functional status. Trp-Kyn metabolism increased in many diseases with chronic immune activation, such as chronic inflammatory, autoimmune, and allergic diseases, as well as cancer and atherosclerosis [10]. Enhanced extrahepatic Trp-Kyn metabolism was reported to be associated with poor prognosis in cancer or disease activity in chronic inflammatory disorders [11, 12]. Collectively, we hypothesized that the activity of Trp-Kyn metabolism reflects the disease severity and may affect the prognosis in DM.

Interestingly, it has been reported that Trp metabolism is significantly altered in DM [13]. However, further clinical information about its downstream Trp-Kyn metabolism is limited. Therefore, we measured the serum levels of Trp and Kyn and calculated the serum Kyn/Trp ratio to determine the association of Trp-Kyn metabolism with clinical characteristics, treatment, and clinical outcomes in DM patients.

## Patients and methods

### Patients

This was a single-center, retrospective, observational study. From January 2017 to September 2020, we enrolled 57 newly diagnosed and treatment-naïve DM patients admitted to the Department of Dermatology at Shanghai Rui Jin Hospital. We also included 10 age- and sex-matched healthy controls (HCs). Classic DM (CDM) or clinically amyopathic DM (CADM) was diagnosed based on Bohan and Peter’s criteria or modified Sontheimer’s definitions [14, 15]. Treatment-naïve DM was defined as having no history of corticosteroid or immunosuppressant use before admission. All patients completed an initial screening before enrollment to evaluate organ involvement and tumor. ILD was diagnosed based on characteristic abnormalities on high-resolution computed tomography (HRCT) and clinical presentations, with the exclusion of pulmonary infections determined by two experienced pulmonologists. All DM patients were divided into two groups according to the optimal cutoff value of the serum Kyn/Trp ratio (× 10^−3^) determined by X-tile software [16], the high serum Kyn/Trp ratio group (> 38.50, *n* = 32) and the low serum Kyn/Trp ratio group (≤ 38.50, *n* = 25). The Ruijin Hospital Ethics Committee approved this study according to Confidential Agreement No. 2016 (105), and informed consent was obtained from all participants.

### Data collection

Patient general characteristics and clinical information were retrieved from hospital electronic medical records by a dedicated person from the Department of Information Technology. All patients were followed up until the endpoint of this study (June 30, 2021), and data were collected without any missing information. Survival time was defined as the time to death or censoring date from the hospital admission date. Electronic medical records were used to collect and collate the corresponding clinical characteristics and laboratory data. Cutaneous Dermatomyositis Disease Area and Severity Index (CDASI) was performed by two dermatologists (H.C. and D.W.), as reported previously [17]. The serum Kyn/Trp ratio was calculated to represent a surrogate marker for Trp-Kyn metabolism level, and X-tile software was used to determine the optimal cutoff value.

### Measurement of serum tryptophan and kynurenine concentrations

Sera were separated by centrifugation at 3000 rpm for 5 min at room temperature and stored frozen at − 80 °C away from light until use. The serum concentrations of Kyn and Trp were quantified using an Agilent 1100 high-performance liquid chromatography-mass spectrometer (HPLC–MS) system. Serum samples were pre-treated using protein precipitation. A 10 µL aliquot of the supernatant was injected into the HPLC–MS system equipped with an Agilent ZORBAX SB-C18 column (2.1 mm × 150 mm, 5 µm) to acquire the mass spectra peak area under the selected ion monitor (SIM) mode. The serum concentrations of Trp and Kyn were evaluated using an internal standard calibration curve. The calibration curve was established as follows. Taking the concentrations of Trp or Kyn calibration samples as the X-axis, the ratio of the mass spectra peak area between Trp or Kyn to the internal standard (metronidazole) as the Y-axis, the regression equation of concentration and mass spectra peak area was obtained using the least squares method.

### Statistical analysis

Continuous variables with normal distribution are reported as the mean ± standard deviation (*SD*). Continuous variables with a skewed distribution are reported as medians with interquartile ranges (*IQR*s). Categorical variables are presented as frequencies (percentages). We used the Student’s *t*-test for continuous variable comparisons or the Mann–Whitney *U* test for non-parametric continuous variable comparisons. Categorical variables were compared using the chi-squared test or Fisher’s exact test. The results were corrected for multiple comparisons using Benjamin-Hochberg false discovery rate (FDR). Spearman’s rank correlation analysis was used for non-parametric correlation analysis. Survival analysis was performed using the Kaplan–Meier method and log-rank test. Cox proportional-hazards regression analysis was used for the assessment of prediction of mortality. The Wilcoxon signed rank-sum test was used to compare the data before and after medication. Statistical significance was set at *P* < 0.05. Statistical analysis and graphing were performed using GraphPad Prism 8 software and R software (R version 4.0.4, http://www.R-project.org).

## Results

### Patient characteristics

In our cohort study, the general information of DM patients is summarized in Supplementary Table 1, including 24 (42.1%) cases of CDM and 33 (57.9%) cases of CADM. The average age of onset was 52.39 ± 15.6 years old (age range 20–79 years). Eighteen (31.6%) were male (9 CDM and 9 CADM), and 39 (68.4%) were female (15 CDM and 24 CADM). Twenty-nine (50.9%) patients had ILD, and 16 (28.1%) had malignancies, including breast, lung, and nasopharyngeal cancers. Among them, CDM patients had a higher incidence of malignancy than those with CADM (21% vs. 7%, respectively; *P* = 0.065).

### Elevated serum Kyn/Trp ratio in dermatomyositis

**Fig. 1:**
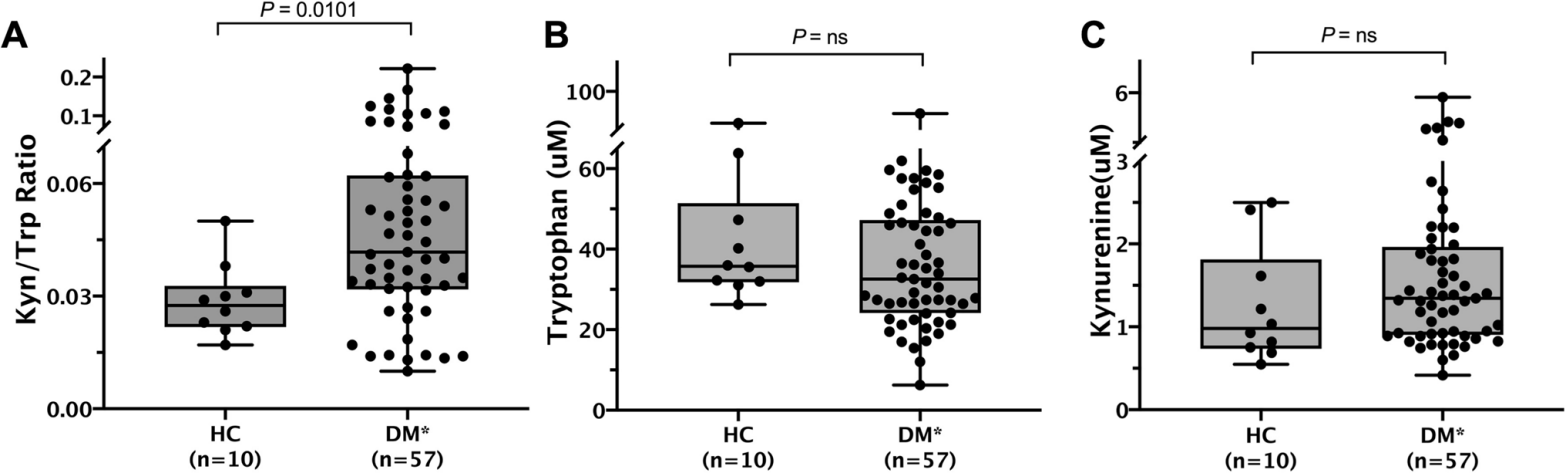
Comparison of serum Kyn/Trp ratio **(A)**, tryptophan **(B)**, and kynurenine **(C)** levels between DM patients and healthy controls. DM* comprises clinically amyopathic dermatomyositis (CADM) and classic dermatomyositis (CDM). Comparisons between two groups were assessed by the Mann–Whitney *U* test. Bars represent medians ± interquartile range. Abbreviations: Kyn/Trp, kynurenine to tryptophan; HC, healthy control; DM, dermatomyositis

The serum Kyn/Trp ratio (× 10^−3^) was significantly higher in all treatment–naïve DM patients than in healthy controls (HCs) (41.73 (31.78–62.15) vs. 27.50 (21.75–32.75), respectively; *P* = 0.0101) (Fig. 1A). In contrast, the serum Trp level (µM) was numerically lower in DM patients compared with HCs (32.55 (24.12–47.21) vs. 35.77 (31.75–51.4), respectively; *P* = 0.2389) (Fig. 1B). The serum Kyn level (µM) was numerically higher in DM patients compared with HCs (1.344 (0.902–1.964) vs. 0.9795 (0.736–1.813), respectively; *P* = 0.1922) (Fig. 1C). Though the differences in tryptophan and kynurenine levels did not reach statistical significance.

### Associations of serum Kyn/Trp ratio with disease activity indices

**Fig. 2:**
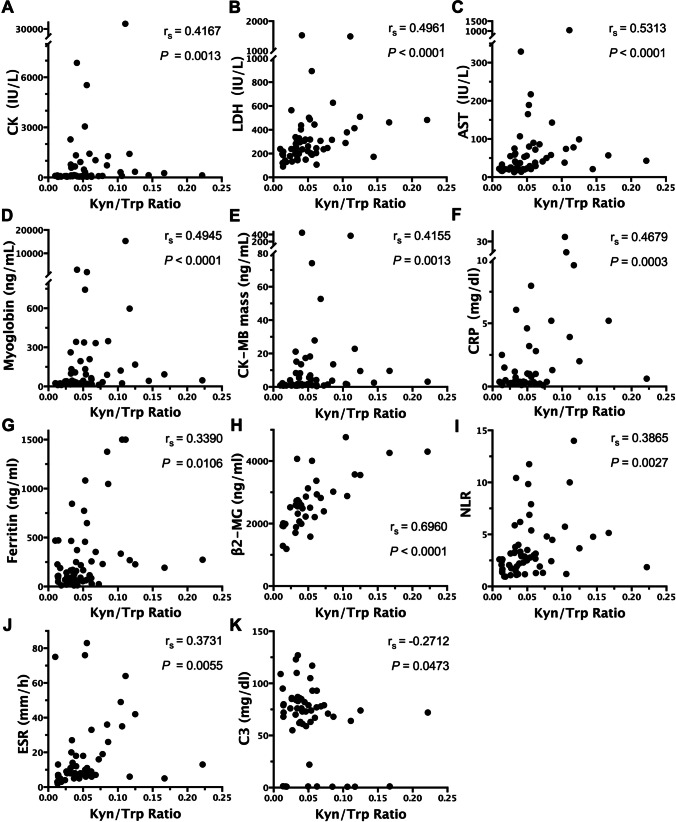
Serum Kyn/Trp ratio correlates with the serological indices in DM patients. **A**–**E** Correlation of serum Kyn/Trp ratio with CK, LDH, AST, myoglobin, and CM-MB mass (*n* = 57). **F**–**K** Correlation of serum Kyn/Trp ratio with CRP (*n* = 55), ferritin (*n* = 56), β2-MG (*n* = 38), NLR (*n* = 57), ESR (*n* = 54), and C3 (*n* = 54). Spearman’s rank correlation analysis was used for non-parametric correlation analysis. Abbreviations: Kyn/Trp, kynurenine to tryptophan; CK, creatine kinase; LDH, lactate dehydrogenase; AST, aspartate aminotransferase; CK-MB, creatine kinase isoenzyme-MB; CRP, C-reactive protein; β2-MG: beta-2-microglobulin; NLR, neutrophil/ lymphocyte ratio; ESR, erythrocyte sedimentation rate; C3, complement C3

In terms of serological indices, the serum Kyn/Trp ratio was found to be positively correlated with muscle enzymes, such as creatine kinase (CK) (*r*_s_ = 0.4167), lactate dehydrogenase (LDH) (*r*_s_ = 0.4961), aspartate aminotransferase (AST) (*r*_s_ = 0.5313), myoglobin (*r*_s_ = 0.4945), and CK isoenzyme-MB (CK-MB) mass (*r*_s_ = 0.4155), and clinical inflammatory indicators, such as C-reactive protein (CRP) (*r*_s_ = 0.4679), serum ferritin (SF) (*r*_s_ = 0.3390), β2-microglobulin (β2-MG) (*r*_s_ = 0.6960), neutrophil/lymphocyte ratio (NLR) (*r*_s_ = 0.3833), and erythrocyte sedimentation rate (ESR) (*r*_s_ = 0.3731). However, the serum Kyn/Trp ratio was negatively correlated with complement C3 (*r*_s_ =  − 0.2712) (Fig. 2A-K). There was no association between the serum Kyn/Trp ratio and CDASI (activity or damage) scoring system.

### Patient characteristics according to serum Kyn/Trp ratio

An optimal cutoff tool named “X-tile software” was used to stratify DM patients into high (serum Kyn/Trp ratio (× 10^−3^) > 38.50, *n* = 32) and low (serum Kyn/Trp ratio (× 10^−3^) ≤ 38.50, *n* = 25) tryptophan metabolism group. Patient key characteristics are listed in Table 1 (complete characteristics are reported in Supplementary Table 3). No statistical differences in age at diagnosis, sex, and body mass index (BMI) were found between the two groups.

**Table 1:** Patient key characteristics according to serum Kyn/Trp ratio

	High Kyn/Trp ratio (*n* = 32)	Low Kyn/Trp ratio (*n* = 25)	*P* value
Anthropometry			
Age, years	54.2 ± 15.9	50.1 ± 15.2	0.615
Female	20 (62.5%)	19 (76.0%)	0.717
BMI, Kg/mm^2^	23.7 (22.2–25.3)	22.7 (22.3–25.1)	0.848
Clinical manifestation			
CADM*	12 (37.5%)	21 (84.0%)	0.008**
CDM*	20 (62.5%)	4 (16.0%)	
ILD	20 (62.5%)	9 (36.0%)	0.224
CIP	14 (43.8%)	8 (32.0%)	
A/SIP	6 (18.8%)	1 (4.00%)	
Malignancy	12 (37.5%)	4 (16.0%)	0.314
Muscle weakness*	20 (62.5%)	5 (20.0%)	0.019*
Antibody			
Anti-TIF1-γ +	9 (28.1%)	10 (40.0%)	0.754
Anti-MDA5 +	7 (21.9%)	3 (12.0%)	0.749
Muscle enzyme			
Creatine kinase, IU/L	214 (93.5–1093)	89.0 (69.0–121)	0.065
LDH*, IU/L	316 (236–467)	198 (144–256)	0.004**
AST*, IU/L	56.5 (35.0–92.2)	23.0 (20.0–36.0)	0.002**
CK-Mb mass*, ng/ml	3.90 (1.37–14.4)	1.20 (0.90–2.20)	0.024*
Myoglobin*, ng/ml	90.6 (28.9–334)	27.4 (18.1–38.3)	0.008**
Routine blood test			
WBC, /L	4.20 (2.97–5.32)	4.50 (4.10–4.87)	0.761
RBC, /L	4.00 ± 0.63	4.26 ± 0.48	0.214
PLT, /L	172 ± 51.5	195 ± 45.7	0.224
Hb*, g/L	121 ± 18.9	132 ± 13.1	0.040*
HCT	0.36 ± 0.05	0.39 ± 0.04	0.080
Neutrophil %*	65.7 ± 11.8	57.0 ± 11.1	0.032*
Neutrophil #, /L	2.50 (2.01–3.86)	2.47 (2.00–2.70)	0.733
Lymphocyte %*	19.8 ± 8.90	28.4 ± 9.92	0.008**
Lymphocyte #*, /L	0.70 (0.50–1.00)	1.30 (0.90–1.70)	0.003***
Basophil %	0.50 (0.30–0.80)	0.60 (0.40–0.70)	0.707
Basophil #, /L	0.00 (0.00–0.01)	0.00 (0.00–0.00)	0.762
Eosinophil %	2.80 (1.10–5.35)	2.80 (2.10–4.30)	0.839
Eosinophil #, /L	0.12 (0.04–0.23)	0.10 (0.10–0.20)	0.828
Monocyte %	9.39 ± 3.78	10.8 ± 3.99	0.409
Monocyte #, /L	0.40 (0.20–0.48)	0.42 (0.30–0.52)	0.224
Inflammatory markers			
NLR*	3.24 (2.48–5.47)	2.06 (1.36–3.17)	0.019*
CRP*, mg/L	0.98 (0.33–3.56)	0.28 (0.22–0.42)	0.041*
ESR, mm/h	11.5 (7.00–31.2)	8.00 (5.75–11.2)	0.141
Ferritin, ng/mL	230 (87.7–415)	105 (66.6–170)	0.096
β2-MG*, ng/mL	3022 ± 823	2247 ± 667	0.019*
Hepatic and renal function			
Albumin*, g/L	33.0 ± 5.95	38.9 ± 3.99	0.002**
Albumin to Globulin*	1.20 ± 0.30	1.47 ± 0.30	0.008**
Prealbumin*, mg/L	158 ± 47.1	226 ± 47.1	0.002**
Total protein*, g/L	61.3 ± 7.25	66.2 ± 6.24	0.041*
γ-GT, IU/L	18.5 (13.0–28.2)	20.0 (15.0–32.0)	0.717
ALT, IU/L	30.5 (21.0–50.2)	20.0 (13.0–30.0)	0.088
Alkaline phosphatase, IU/L	58.5 (51.0–78.5)	64.0 (51.0–74.0)	0.918
Blood lipids			
Total cholesterol*, mmol/L	3.92 ± 0.95	4.73 ± 1.06	0.028*
LDL, mmol/L	2.38 ± 0.79	2.83 ± 0.67	0.096
Triglycerides, mmol/L	1.48 (1.21–1.81)	1.52 (1.26–2.29)	0.754
HDL, mmol/L	0.94 ± 0.33)	1.13 ± 0.28	0.080
Free fatty acids, mmol/L	0.45 (0.35–0.57)	0.46 (0.32–0.48)	0.548
ApoAI*, g/L	1.01 ± 0.27)	1.25 ± 0.19	0.002**
ApoB, g/L	0.82 ± 0.22)	0.86 ± 0.20	0.743
ApoE, mg/dL	4.35 (3.68–5.03)	4.30 (3.50–5.75)	0.918
Lipoprotein(a), g/L	0.10 (0.06–0.21)	0.18 (0.11–0.21)	0.224
Electrolytes			
Calcium*, mmol/L	2.07 ± 0.15	2.22 ± 0.12	0.002**
Potassium, mmol/L	3.85 ± 0.35	3.94 ± 0.29	0.524
Sodium, mmol/L	141 (139–142)	141 (139–142)	0.761
Tumor markers			
NSE*, ng/mL	21.7 (15.6–32.6)	14.5 (11.9–16.4)	0.008**
CA153*, U/mL	13.2 (9.70–20.0)	9.30 (6.38–10.9)	0.015**

Continuous variables with normal distribution: mean ± standard deviation (SD); continuous variables with skewed distribution: median (interquartile range, *IQR*); categorical variables: frequencies (percentages), *n* (%)

*IDO1*, indoleamine 2,3-dioxygenase 1; *BMI*, body mass index; *CADM*, clinically amyopathic dermatomyositis; *CDM*, classic dermatomyositis; *ILD*, interstitial lung disease; *CIP*, chronic interstitial pneumonia; *A/SIP*, acute/subacute interstitial pneumonia; *TIF1-γ*, transcription intermediary factor 1-gamma; *MDA5*, melanoma differentiation-associated protein 5; *CK*, creatine kinase; *LDH*, lactate dehydrogenase; *AST*, aspartate aminotransferase; *CK-MB*, creatine kinase isoenzyme-MB; *WBC*, white blood cell count; *RBC*, red blood cell count; *PLT*, platelet count; *Hb*, hemoglobin; *HCT*, hematocrit; *NLR*, neutrophil/lymphocyte ratio; *CRP*, C-reactive protein; *ESR*, erythrocyte sedimentation rate; *β2-MG*, beta-2-microglobulin; *γ-GT*, gamma-glutamyltransferase; *ALT*, alanine aminotransferase; *LDL*, low-density lipoprotein; *HDL*, high-density lipoprotein; *ApoAI*, apolipoprotein A-I; *NSE*, neuro-specific enolase; *CA153*, carbohydrate antigen 153; *APTT*, activated partial thromboplastin time; *Fg*, fibrinogen; *INR*, international normalized ratio; *PT*, prothrombin time; *TT*, thrombin time

*, *P* < 0.05; **, *P* < 0.01; ***, *P* < 0.001. *P*-values were adjusted using Benjamin-Hochberg false discovery rate (FDR)

DM patients with high serum Kyn/Trp ratio had a significantly higher frequency of CDM (62.5% vs. 16%, *P* = 0.008) and a lower frequency of CADM (37.5% vs. 84.0%, *P* = 0.008) than those with low serum Kyn/Trp ratio, in concert with a higher percentage of muscle weakness symptoms (62.5% vs. 20.0%, *P* = 0.019) and higher levels of LDH (316.0 (236.0–467.0) vs. 198.0 (144.0–256.0), *P* = 0.004), AST (56.5 (35.0–92.2) vs*.* 23.0 (20.0–36.0), *P* = 0.002), CK-MB (3.9 (1.4–14.4) vs. 1.2 (0.9–2.2), *P* = 0.024), and myoglobin (90.6 (28.9–334.0) vs. 27.4 (18.1–38.3), *P* = 0.008).

Additionally, DM patients with high serum Kyn/Trp ratio had higher levels of inflammatory markers than those with low serum Kyn/Trp ratio, including NLR (3.2 (2.5–5.5) vs. 2.1 (1.4–3.2), *P* = 0.019), CRP (1.0 (0.3–3.6) vs. 0.3 (0.2–0.4), *P* = 0.041), and β2-MG (3022 ± 823 vs. 2247 ± 667, *P* = 0.019).

### Survival analysis according to serum Kyn/Trp ratio

**Fig. 3:**
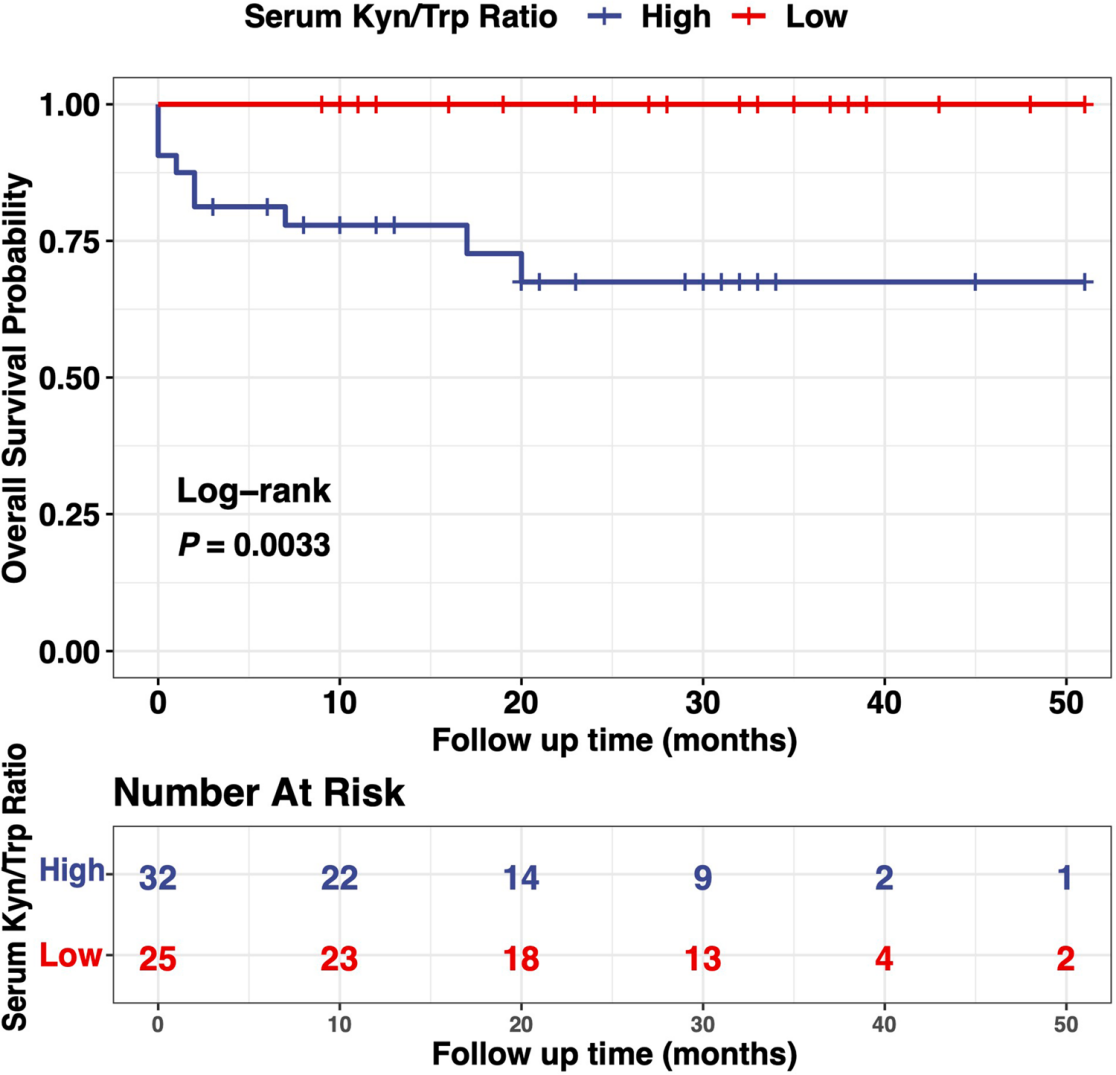
Survival curves according to the optimal cutoff for serum Kyn/Trp ratio in all DM patients. Survival analysis (*n* = 57) was performed by the Kaplan Meier method and log-rank test. Abbreviations: IDO1, indoleamine 2,3-dioxygenase 1; DM, dermatomyositis

In our cohort, the cumulative survival rate was 86.19%. Survival analysis showed that DM patients with high Trp-Kyn metabolism had worse overall survival than those with low Trp-Kyn metabolism (log-rank *P* = 0.0033) (Fig. 3). Patients with high Trp-Kyn metabolism had a higher frequency of comorbidities, including interstitial lung disease or malignancy, and the main causes of death were respiratory failure due to interstitial lung disease (3/9, 33.3%) or the exacerbation of malignancy (6/9, 66.7%). Cox proportional-hazards regression model showed that ln(Kyn/Trp) was an independent predictor of mortality in the cohort under investigation. After adjusting for BMI, WBC, malignancy, hypertension, and diabetes mellitus, ln(Kyn/Trp) (HR 4.874, 95% *CI* 1.105–21.499, *P* = 0.036) was still an independent predictor of mortality (Table 2).

**Table 2:** Results of multivariate Cox regression analyses of ln(Kyn/Trp) in patients with dermatomyositis

Variable	HR	95%*CI*	*P* value
**ln(Kyn/Trp)**	4.874	1.105–21.499	0.036*
BMI	1.138	0.953–1.359	0.152
WBC	0.783	0.463–1.323	0.360
Malignancy	2.163	0.461–10.147	0.328
Hypertension	0.121	0.010–1.474	0.123
Diabetes mellitus	6.889	1.117–42.469	0.060

### Impact of dermatomyositis treatment on serum Kyn/Trp ratio

**Fig. 4:**
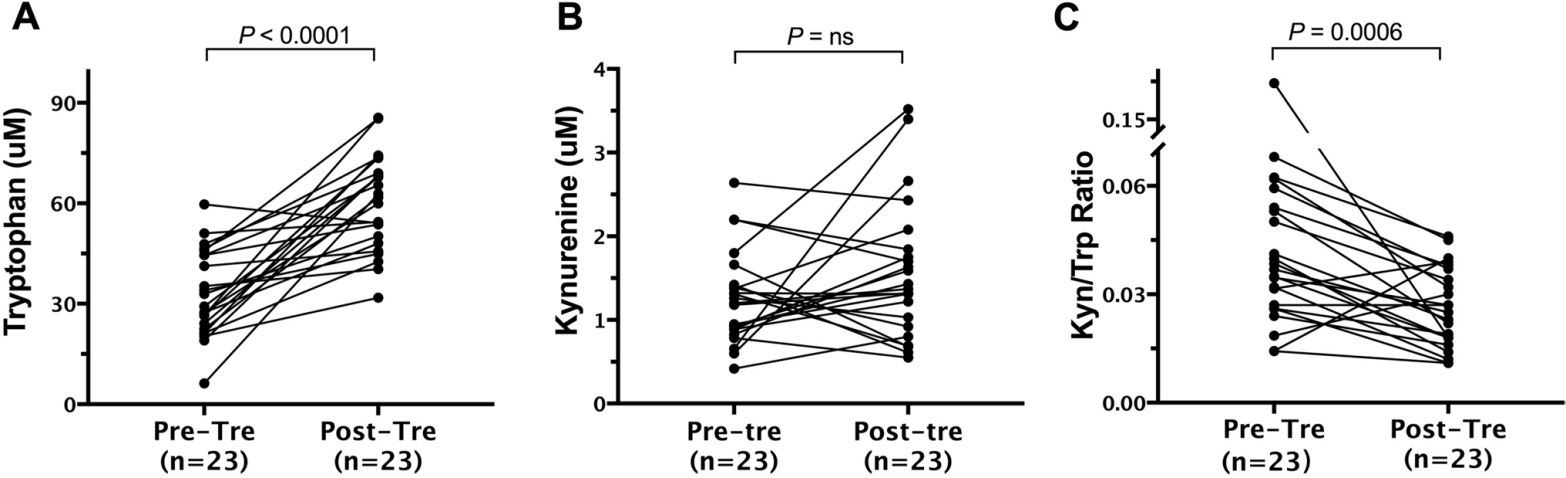
Serum tryptophan and kynurenine levels as well as Kyn/Trp ratio before and after treatment in DM patients (*n* = 23). The Wilcoxon signed rank-sum test was used to compare the data before and after medication. Abbreviations: Pre-Tre, pre-treatment; Post-Tre, post-treatment; Kyn/Trp, kynurenine to tryptophan

The effect of DM medication on the serum Kyn/Trp ratio was assessed in 23 individuals that achieved disease remission with serum samples available during follow-up. The mean duration of treatment was 3.30 ± 1.52 months, ranging from 1 to 7 months. Therapeutic strategies for DM are summarized in Supplementary Table 2. After comprehensive treatment, the serum Kyn/Trp ratio decreased significantly (36.89 (26.00–54.00) vs. 25.00 (18.00–37.00), *P* = 0.0006) (Fig. 4A), and the level of serum Trp (µM) concentration increased significantly (29.19 (22.44–44.55) vs. 61.76 (48.05–68.98), *P* < 0.0001) (Fig. 4B). However, the changes in serum Kyn levels were not statistically significant (*P* = 0.2467, Fig. 4C).

## Discussion

The essential role of immunity is “self–nonself” discrimination [18]. Inflammation can be concluded as the activation of immune cells during the defense and clearance of “nonself.” However, they were always limited and restrained by intrinsic immune regulation function in a feedback loop to restore immune homeostasis. Trp-Kyn metabolism level is an important indicator for such intrinsic immune functional status. In this study, we explored the clinical association of the Trp-Kyn metabolism via IDO1 induction in DM and demonstrated that (1) an increased serum Kyn/Trp ratio in DM correlated with disease activity; (2) Cox regression analyses identified ln(Kyn/Trp) as an independent prognostic predictor of mortality; and (3) serum Kyn/Trp ratio decreased upon treatment.

In previous basic research, it has been identified that higher Trp oxidation in the muscle of patients with polymyositis contributes to the mitochondrial proteins’ alteration and mitochondrial dysfunction, which is a common pathway shared in muscle disease [19]. Later, a bio-informatic metabolomic study identified the aminoacyl-tRNA biosynthesis, Trp biosynthesis, and metabolism were perturbed pathways in DM and showed that the decreased Trp levels had diagnostic values for DM [13]. In this study, the clinical aspect of Trp metabolism in DM was considered through the disease activity, treatment, and prognosis.

Trp-Kyn metabolism via IDO1 induction has also been reported with clinical relevance in other autoimmune diseases. For example, in SLE patients, enhanced Trp-Kyn metabolism correlated with disease activity and severe fatigue and could predict disease activation [20–23]. Elevated IDO1 activity and increased regulatory T cells have been discovered and correlated with disease severity and interferon-positive signatures in patients with Sjogren’s syndrome [24, 25]. Moreover, serum IDO1 activity was confirmed to be increased in psoriasis, where immune cells from patients with psoriasis are defective in inducing IDO1 upregulation in response to inflammatory stimuli [26].

In this study, we observed that patients with high Kyn/Trp ratio had more muscle involvement (CADM vs. CDM, muscle enzymes), which is in congruence with previous basic research, indicating that an enhanced extrahepatic Trp-Kyn metabolic pathway and implying that the subsequent intrinsic immune regulation function could play a role in muscle tissue damage and repairment. It is reasonable to speculate that Trp-Kyn metabolism may also contribute to the robustness of the tissue reconstruction system by maintaining immune homeostasis [27]. Skeletal muscle is the largest reservoir of amino acids in the body; it has been reported that aminoacyl-tRNA, phenylalanine, and tyrosine biosynthesis pathways were also altered in DM. Muscle metabolism–related changes in immune function may be a new therapeutic target for DM.

What’s more, the serum Kyn/Trp ratio correlated with disease activity indicators. However, there was no correlation between the serum Kyn/Trp ratio and CDASI (activity or damage). We hypothesized that the CDASI indicates the severity of skin lesions while the Kyn/Trp ratio represents the whole level of immune status. Given the alterations of Trp-Kyn metabolism before and after treatment, the level of ln(Kyn/Trp) showed potential prognostic value for survival. This would implicate the underlying impairment in the intrinsic immune regulation function in patients with DM.

**Fig. 5:**
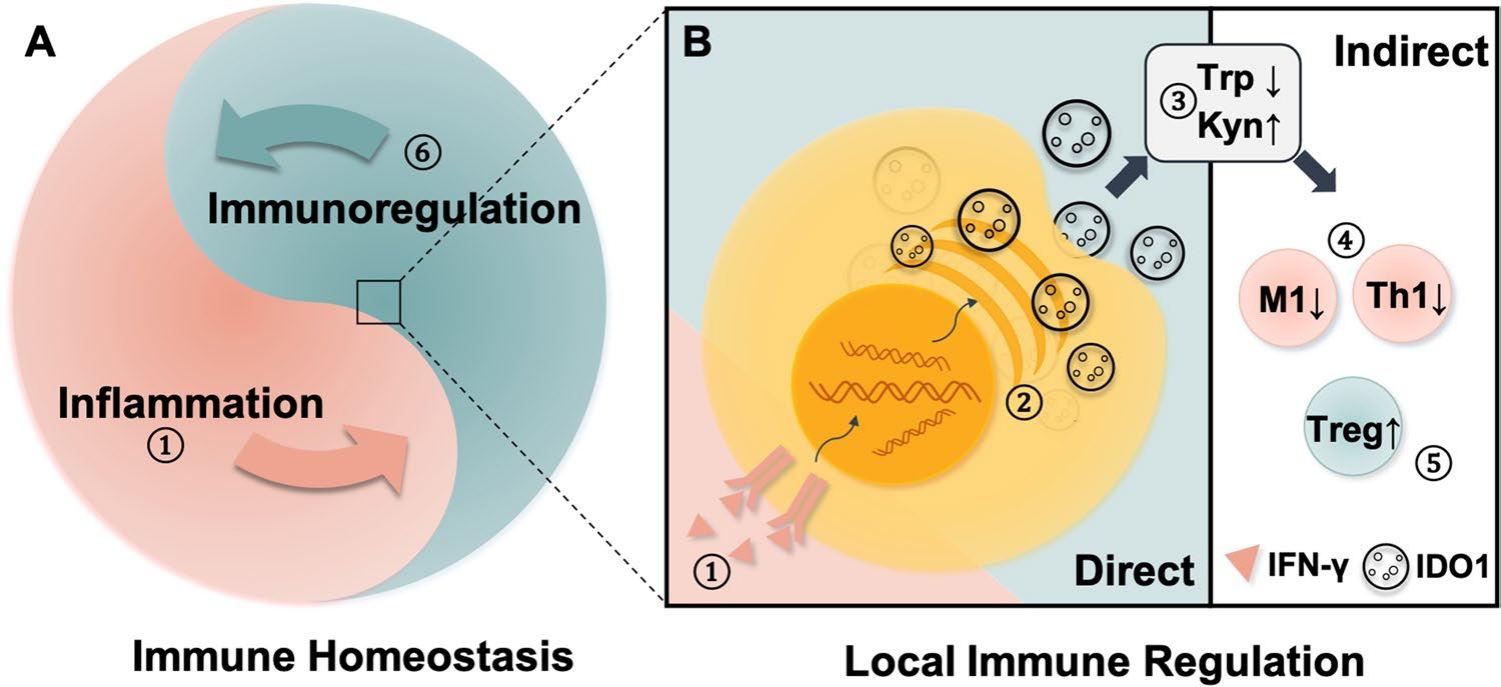
Immune homeostasis and local immune regulation via the tryptophan-kynurenine metabolic pathway via IDO1 induction. **A** Inflammation and immune regulation co-occur to maintain dynamic immune homeostasis. **B** IDO1 induction upon inflammatory stimuli drives the extrahepatic tryptophan-kynurenine metabolic pathway to play a role in local immune regulation. ① Pro-inflammatory stimuli; ② IDO1 induction; ③ enhanced extrahepatic tryptophan-kynurenine pathway; ④ altered phenotype of immune cells; ⑤ immune tolerance and immune suppression; ⑥ immune homeostasis. Abbreviations: Trp, tryptophan; Kyn, kynurenine; IFN-γ, interferon-gamma; IDO1, indoleamine 2,3-dioxygenase 1; M1, M1 macrophages; Th1, CD4 + T helper 1; Treg, regulatory T cell

Although this is an observational study, this research has important implications. Once inflammation was initiated, the intrinsic immune regulation function also occurred (Fig. 5A). It may be driven by extrahepatic Trp-Kyn metabolism via IDO1 induction to limit the excessive inflammatory reactions (Fig. 5B) [28]. Such findings can be explained as “Trp depletion” and “Kyn production” [29]. The former leads to the inhibition of effector immune cells via the general control nonderepressible two kinase (GCN2K) signaling pathway. The latter activates the aromatic hydrocarbon receptor (AhR) signaling pathway, which induces anti-inflammatory signaling [30]. A Trp-Kyn metabolism via the IDO1 induction comic is shown in Supplementary Fig. 1. So we proposed that other than “anti-inflammation” therapeutic strategies, intrinsic immune regulation function would be a promising pipeline for the treatment and surveillance of DM.

The current first-line treatment of DM is still mainly empirical glucocorticoids with immunosuppressants [31]; due to its side-effects, new approaches are still in need. Trp-Kyn metabolism could be an essential target for restoration of intrinsic immune regulation function with potentially translational implications and may be a source of future work. First, regular aerobic exercise of a certain intensity can promote muscle recovery by altering the Kyn pathway [32]. Intensive aerobic exercise and resistance training can reduce disease activity and promote muscular improvement in DM [33, 34]. Shifting the Kyn pathway after exercise could be one reason that non-pharmacological treatment exercises effectively treat DM. Second, changes in food composition and lifestyle, including physical activity and weight loss, have been shown to influence Trp availability [35, 36]. The regulation of Trp content showed clinical potential in the prevention and treatment of immune-related disorders, which may also have therapeutic potential in DM [37]. Recently, IDO1 inhibitors have been developed and entered the clinical assessment phase and show promise for cancer therapy by strengthening the anti-tumor immune response [38]. Regulating IDO1 expression and activity may be a potential therapeutic strategy for DM.

Overall, we added additional evidence to the literature pool on recognizing another potential marker for clinical outcome prediction among patients with DM. A better understanding of such a signaling pathway could help physicians to differentiate between the DM subtypes. More importantly, our study showed that intrinsic immune regulation function is also of paramount importance for immune homeostasis and could also be a target for therapy in DM.

Importantly, our investigation has some limitations that require discussion. First, the presented data demonstrate associations rather than causal relationships or mechanistic insights. Second, without directly assessing IDO1 expression in serum, we can only hypothesize that increased Trp-Kyn metabolism in DM may associate with altered intrinsic immune regulation function. Interventional clinical studies are needed to determine whether modification of intestinal tryptophan pathways affects the severity of DM. Third, the sample size of our investigation is rather limited. Further studies with larger sample sizes and prospective cohorts are required to confirm our findings. Fourth, other disease-specific outcome measures such as physician global disease activity scores, manual muscle testing, or patient reported outcomes had not been done to comprehensively evaluate the severity of the disease.

In the present study, we determined an immunometabolic feature of enhanced Trp-Kyn metabolism and its clinical relevance in treatment-naïve adult DM patients. The role of immune regulation via the Trp-Kyn pathway triggered by IDO1 induction in DM needs to be further investigated for its clinical potential in the future. Monitoring and intervention in the functional state of immune regulation in the human body need to be considered as a potential therapeutic target in DM.

## Supplementary Information

Below is the link to the electronic supplementary material.

Supplementary file1 (DOCX 2783 KB)

## Data Availability

All data of this study are available in the article or uploaded as online supplementary material.
