# Correction to: Enhanced tryptophan-kynurenine metabolism via indoleamine 2,3-dioxygenase 1 induction in dermatomyositis

**DOI:** 10.1007/s10067-022-06317-6

**Published:** 2022-07-28

**Authors:** Dan Wu, Mengya Chen, Shile Chen, Shimin Zhang, Yongheng Chen, Qian Zhao, Ke Xue, Feng Xue, Xiaosong Chen, Min Zhou, Hao Li, Jie Zheng, Yunchen Le, Hua Cao

**Affiliations:** 1 Department of Dermatology, Rui Jin Hospital, Shanghai Jiao Tong University School of Medicine, No. 197, Rui Jin 2nd Road, Shanghai, 200025 China; 2 Comprehensive Breast Health Center, Rui Jin Hospital, Shanghai Jiao Tong University School of Medicine, Shanghai, China; 3 Department of Respiratory and Critical Care Medicine, Rui Jin Hospital, Shanghai Jiao Tong University School of Medicine, Shanghai, China; 4 Department of Oncology, Rui Jin Hospital, Shanghai Jiao Tong University School of Medicine, Shanghai, China

**Correction to: Clinical Rheumatology**

**https://doi.org/10.1007/s10067-022-06263-3**

The original published version of the above article contained incorrect Key Points. The authors requested to note the following corrected points.

The Key Points of the article should be:

Key Points:

1. HPLC-MS identified increased serum Kyn/Trp ratio in DM patients, which positively correlated with levels of muscle enzymes and inflammatory markers and was downregulated upon treatment.

2. Cox regression analyses identified ln(Kyn/Trp) as an independent prognostic predictor of mortality in DM.

3. Monitoring intrinsic immune regulation function should be considered a potential therapeutic target in DM patients.

Instead of:

Key Points:

1. HPLC–MS identified increased serum Kyn/Trp ratio in DM patients, which positively correlated with levels of muscle enzymes and inflammatory markers and was downregulated upon treatment.

2. More attention to psychological status is needed in RA patients.

3. Cox regression analyses identified ln(Kyn/Trp) as an independent prognostic predictor of mortality in DM.

The original article has been corrected.

