# Supplementary material for: Enhanced tryptophan-kynurenine metabolism via indoleamine 2,3-dioxygenase 1 induction in dermatomyositis
Source: Clin Rheumatol. 2022 Jul 1;41(10):3107–17. doi: 10.1007/s10067-022-06263-3 (PMC9485101; doi:10.1007/s10067-022-06263-3)
Supplement: Supplementary file 1 — Supplementary file1 (DOCX 2783 KB) [file 10067_2022_6263_MOESM1_ESM.docx]

**Supplementary Data**

**Enhanced tryptophan-kynurenine metabolism via indoleamine 2,3-dioxygenase 1 induction in dermatomyositis**

**Running Head: Tryptophan-kynurenine metabolism via IDO1 in DM**

Dan Wu^1^, Mengya Chen^1^, Shile Chen^1^, Shimin Zhang^1^, Yongheng Chen^1^, Qian Zhao^1^, Ke Xue^1^, Feng Xue^1^, Xiaosong Chen^2^, Min Zhou^3^, Hao Li^4^, Jie Zheng^1,5^, Yunchen Le^1,5^, and Hua Cao^1,5^

**Table of content**

1. **Figures**
2. ***Supplementary Figure 1***
3. **Tables**
4. ***Supplementary Table 1***
5. ***Supplementary Table 2***
6. ***Supplementary Table 3***

***Supplementary Figure 1***

**A tryptophan (Trp)-kynurenine (Kyn) metabolism via indoleamine 2,3-dioxygenase 1 (IDO1) induction comic.**

In this cartoon world,

[A]. We analogize Trp to a supply in the energy supermarket where is exogenous and available in limited quantities. The guarantee of Trp supply would be significant because they allow the immune cells to thrive otherwise to die. However, the IDO1 babies are "Trp addicts".

[B]. We analogize Kyn to smelly products excreted by IDO1 babies after intoxicating themselves with Trp. The production of Kyn are also poisons of over-active immune cells.

[C]. The occurrence of inflammatory stimulations called gene factories to produce IDO1. The newborn IDO1 babies are so excited about consuming Trp. (IDO1 induction)

[D]. The IDO1 babies wipe out the Trp reserves in the energy market, leaving the effector immune cells too "hungry" to die. (Trp depletion)

[E]. Ingested Trp ferment in IDO1's stomach and then turned into Kyn. IDO1 babies feel nauseous and vomit up the contents of their stomachs which kill immune cells simultaneously. (Kyn production)

[F]. In summary, the IDO1 induction upon inflammatory stimulations leads to Trp depletion and Kyn production, which exerts an essential role in immune regulation. (Enhanced Trp-Kyn metabolism via IDO1 induction)

**Supplementary Table 1.** Patient Characteristics.

| **Characteristic** | **All DM (n = 57)** | **CDM (n = 24)** | **CADM (n = 33)** | ***P* value** |
| --- | --- | --- | --- | --- |
| Female | 39 (68%) | 15 (62%) | 24 (73%) | 0.719 |
| Age of onset, years | 52.39 ± 15.60 | 57.25 ± 13.82 | 48.85 ± 16.06 | 0.094 |
| BMI | 23.51  (22.27-25.14) | 24.05  (22.94-25.94) | 22.59  (21.88-24.61) | 0.162 |
| **Clinical characteristics** | | | | |
| Pruritus | 26 (46%) | 8 (33%) | 18 (55%) | 0.339 |
| ILD | 29 (51%) | 14 (58%) | 15 (45%) | 0.682 |
| Malignancy | 16 (28%) | 12 (50%) | 4 (12%) | 0.015* |
| Heliotrope sign | 36 (63%) | 14 (58%) | 22 (67%) | 0.811 |
| Gottron’s papules | 30 (53%) | 11 (46%) | 19 (58%) | 0.685 |
| Gottron’s sign | 15 (26%) | 4 (17%) | 11 (33%) | 0.459 |
| Poikiloderma | 10 (18%) | 5 (21%) | 5 (15%) | 0.811 |
| V-neck sign | 34 (60%) | 15 (62%) | 19 (58%) | 0.920 |
| Shawl sign | 22 (39%) | 10 (42%) | 12 (36%) | 0.920 |
| Skin ulcers | 5 (9%) | 4 (17%) | 1 (3%) | 0.292 |
| Periungual erythema | 30 (53%) | 15 (62%) | 15 (45%) | 0.508 |
| Muscle weakness | 25 (44%) | 19 (79%) | 6 (18%) | 0.001** |
| Dysphagia | 11 (19%) | 8 (33%) | 3 (9%) | 0.094 |
| **Myositis specific antibodies** | | | | |
| Anti-TIF1-γ | 19 (33%) | 9 (38%) | 10 (30%) | 0.833 |
| Anti-MDA5 | 10 (18%) | 3 (12%) | 7 (21%) | 0.682 |
| Anti-SRP | 1 (2%) | 1 (4%) | 0 | - |
| Anti-SAE | 2 (4%) | 0 | 2 (6%) | - |
| Anti-Mi-2 | 2 (4%) | 2 (8%) | 0 | - |
| Anti-Jo1 | 1 (2%) | 0 | 1 (3%) | - |
| Anti-EJ | 2 (4%) | 1 (4%) | 1 (3%) | - |
| Anti-OJ | 1 (2%) | 1 (4%) | 0 | - |
| **Laboratory** | | | | |
| ANAs | 15 (29%) | 8 (36%) | 7 (24%) | 0.685 |
| ENAs | 6 (12%) | 4 (18%) | 2 (7%) | 0.585 |
| CK, IU/L | 112.00  (77.00-587.00) | 747.50  (314.75-1416.50) | 82.00  (57.00-109.00) | 0.001** |
| LDH, IU/L | 247.00  (191.00-378.00) | 370.50  (293.50-490.50) | 202.00  (157.00-247.00) | 0.001** |
| AST, IU/L | 38.00  (21.00-72.00) | 76.50  (50.50-116.00) | 23.00  (20.00-37.00) | 0.001** |
| ALT, IU/L | 25.00  (17.00-41.00) | 34.50  (24.50-55.50) | 20.00  (13.00-30.00) | 0.005** |
| NLR | 2.78  (1.75-4.47) | 4.07  (2.61-6.37) | 2.30  (1.31-3.17) | 0.005** |
| CRP, mg/L | 0.37  (0.24-1.40) | 0.98  (0.31-3.56) | 0.30  (0.22-0.82) | 0.070 |
| ESR, mm/h | 8.50  (6.25-18.00) | 10.00  (8.00-31.00) | 8.00  (6.00-13.50) | 0.141 |
| Ferritin, ng/ml | 164.95  (68.77-340.08) | 269.60  (139.30-552.30) | 105.40  (57.20-188.60) | 0.012* |
| β2-MG, ng/ml | 2674.95 ± 843.28 | 3047.22 ± 885.05 | 2339.90 ± 659.16 | 0.029* |
| **Other comorbidities** | | | | |
| Hypertension | 13 (23%) | 6 (25%) | 7 (21%) | 0.736 |
| Diabetes mellitus | 7 (12%) | 4 (17%) | 3 (9%) | 0.390 |


1. *, *P* < 0.05; **, *P* < 0.01; ***, *P* < 0.001. *P*-values were adjusted using Benjamin-Hochberg false discovery rate (FDR).

2. Abbreviations: CADM, clinically amyopathic dermatomyositis; CDM, classic dermatomyositis; BMI, body mass index; ILD, interstitial lung disease; ANAs, anti-nuclear antibodies; ENAs, anti-extractable nuclear antigen antibodies; TIF1-γ, transcription intermediary factor 1-gamma; MDA5, melanoma differentiation-Associated protein 5; CK, creatine kinase; LDH, lactate dehydrogenase; AST, aspartate aminotransferase; ALT, alanine aminotransferase; NLR, neutrophil/ lymphocyte ratio; CRP, C-reactive protein; ESR, erythrocyte sedimentation rate; β2-MG, beta-2-microglobulin.

3. Continuous variables with normal distribution: mean ± standard deviation (SD); Continuous variables with skewed distribution: median (interquartile range, IQR); Categorical variables: frequencies (percentages), n (%).

**Supplementary Table 2.** Therapeutic strategies for DM.

| **Age of onset** | **Gender** | **Disease** | **Duration of treatment** | **Glucocorticoids** | **IVIG** | **Cytotoxic drugs (Immunosuppressants)** | **Other Drugs (Immunomodulators)** |
| --- | --- | --- | --- | --- | --- | --- | --- |
| 70 | Female | CADM | 3 | / | / | / | TGP |
| 22 | Female | CADM | 4 | Pred | / | / | HCQ, TGP |
| 67 | Male | CDM | 3 | Pred | / | CTX | HCQ, TGP |
| 69 | Female | CADM | 7 | / |  | / | HCQ, TGP |
| 45 | Male | CADM | 4 | Pred | IVIG | CTX | HCQ, TGP, Thalidomide |
| 53 | Female | CDM | 3 | MP, Pred | / | / | HCQ, TGP, Thalidomide |
| 48 | Male | CADM | 6 | / | / | / | HCQ, TGP, Thalidomide |
| 52 | Female | CDM | 2 | MP, Pred | IVIG | MTX | HCQ, TGP, Thalidomide |
| 54 | Male | CDM | 2 | MP, Pred | / | / | / |
| 55 | Female | CADM | 4 | / | / | / | HCQ, TGP |
| 79 | Male | CADM | 1 | / | / | / | TGP |
| 46 | Female | CDM | 3 | MP, Pred | / | / | / |
| 71 | Female | CDM | 2 | MP, Pred | IVIG | / | HCQ, TGP, Thalidomide |
| 20 | Female | CADM | 5 | / | / | / | HCQ, TGP, Baricitinib |
| 47 | Female | CADM | 3 | Pred | / | / | HCQ, TGP |
| 57 | Female | CADM | 2 | MP, Pred | IVIG | CTX | TGP |
| 28 | Male | CADM | 5 | / | / | / | HCQ, TGP |
| 78 | Female | CADM | 2 | / | / | / | HCQ, TGP |
| 60 | Male | CDM | 1 | Pred | IVIG | / | HCQ, TGP |
| 52 | Male | CADM | 4 | Pred | / | / | HCQ, TGP, Thalidomide |
| 41 | Female | CDM | 4 | MP, Pred | / | CTX, CsA | HCQ, TGP, Thalidomide |
| 74 | Female | CADM | 4 | Pred | / | / | HCQ, TGP |
| 40 | Female | CADM | 2 | / | / | / | HCQ, TGP |

Abbreviations: MSA, myositis-specific antibody; TIF, transcription intermediary factor 1-gamma; MDA5, melanoma differentiation-Associated protein 5; CDM, classic dermatomyositis; CADM, clinically amyopathic dermatomyositis; Pred, prednisone; MP, methylprednisone; IVIG, intravenous immunoglobulin; CTX, cyclophosphamide; CsA, Cyclosporine A; HCQ, hydroxychloroquine; TGP, total glucosides of paeony.

**Supplementary Table 3.** Patient complete characteristics according to serum Kyn/Trp ratio.

|  | **High Kyn/Trp ratio (n=32)** | **Low Kyn/Trp ratio (n=25)** | ***P* value** |
| --- | --- | --- | --- |
| **Anthropometry** |  |  |  |
| Age, years | 54.2 ± 15.9 | 50.1 ± 15.2 | 0.615 |
| Female | 20 (62.5%) | 19 (76.0%) | 0.717 |
| BMI, Kg/mm^2^ | 23.7 (22.2-25.3) | 22.7 (22.3-25.1) | 0.848 |
| **Clinical manifestation** |  |  |  |
| **CADM*** | 12 (37.5%) | 21 (84.0%) | **0.008**** |
| **CDM*** | 20 (62.5%) | 4 (16.0%) |  |
| ILD | 20 (62.5%) | 9 (36.0%) | 0.224 |
| CIP | 14 (43.8%) | 8 (32.0%) |  |
| A/SIP | 6 (18.8%) | 1 (4.00%) |  |
| Malignancy | 12 (37.5%) | 4 (16.0%) | 0.314 |
| Heliotrope sign | 17 (53.1%) | 19 (76.0%) | 0.314 |
| Gottron's papules | 17 (53.1%) | 13 (52.0%) | 1.000 |
| Gottron's sign | 10 (31.2%) | 5 (20.0%) | 0.754 |
| Poikiloderma | 7 (21.9%) | 3 (12.0%) | 0.749 |
| V-neck sign | 18 (56.2%) | 16 (64.0%) | 0.848 |
| Shawl sign | 15 (46.9%) | 7 (28.0%) | 0.498 |
| Skin ulcers | 4 (12.5%) | 1 (4.00%) | 0.689 |
| Periungual erythema | 18 (56.2%) | 12 (48.0%) | 0.848 |
| **Muscle weakness*** | 20 (62.5%) | 5 (20.0%) | **0.019*** |
| Dysphagia | 7 (21.9%) | 4 (16.0%) | 0.848 |
| Pruritus | 14 (43.8%) | 12 (48.0%) | 0.979 |
| **Antibody** |  |  |  |
| Anti-TIF1-γ + | 9 (28.1%) | 10 (40.0%) | 0.754 |
| Anti-TIF1-γ titer | 25.2 (2.08-83.2) | 24.6 (3.52-72.9) | 0.919 |
| Anti-MDA5 + | 7 (21.9%) | 3 (12.0%) | 0.749 |
| Anti-MDA5 titer | 1.57 (1.17-73.8) | 2.07 (1.35-7.44) | 0.897 |
| ANA+ | 9 (30.0%) | 6 (28.6%) | 1.000 |
| ENA+ | 3 (10.0%) | 3 (14.3%) | 0.839 |
| **Muscle Enzyme** |  |  |  |
| Creatine kinase, IU/L | 214 (93.5-1093) | 89.0 (69.0-121) | 0.065 |
| **LDH*, IU/L** | 316 (236-467) | 198 (144-256) | **0.004**** |
| **AST*, IU/L** | 56.5 (35.0-92.2) | 23.0 (20.0-36.0) | **0.002**** |
| **CK-Mb mass*, ng/ml** | 3.90 (1.37-14.4) | 1.20 (0.90-2.20) | **0.024*** |
| **Myoglobin*, ng/ml** | 90.6 (28.9-334) | 27.4 (18.1-38.3) | **0.008**** |
| **Routine Blood Test** |  |  |  |
| WBC, /L | 4.20 (2.97-5.32) | 4.50 (4.10-4.87) | 0.761 |
| RBC, /L | 4.00 ± 0.63 | 4.26 ± 0.48 | 0.214 |
| PLT, /L | 172 ± 51.5 | 195 ± 45.7 | 0.224 |
| **Hb*, g/L** | 121 ± 18.9 | 132 ± 13.1 | **0.040*** |
| HCT | 0.36 ± 0.05 | 0.39 ± 0.04 | 0.080 |
| **Neutrophil %*** | 65.7 ± 11.8 | 57.0 ± 11.1 | **0.032*** |
| Neutrophil #, /L | 2.50 (2.01-3.86) | 2.47 (2.00-2.70) | 0.733 |
| **Lymphocyte %*** | 19.8 ± 8.90 | 28.4 ± 9.92 | **0.008**** |
| **Lymphocyte #*, /L** | 0.70 (0.50-1.00) | 1.30 (0.90-1.70) | **0.003***** |
| Basophil % | 0.50 (0.30-0.80) | 0.60 (0.40-0.70) | 0.707 |
| Basophil #, /L | 0.00 (0.00-0.01) | 0.00 (0.00-0.00) | 0.762 |
| Eosinophil % | 2.80 (1.10-5.35) | 2.80 (2.10-4.30) | 0.839 |
| Eosinophil #, /L | 0.12 (0.04-0.23) | 0.10 (0.10-0.20) | 0.828 |
| Monocyte % | 9.39 ± 3.78 | 10.8 ± 3.99 | 0.409 |
| Monocyte #, /L | 0.40 (0.20-0.48) | 0.42 (0.30-0.52) | 0.224 |
| **Inflammatory markers** |  |  |  |
| **NLR*** | 3.24 (2.48-5.47) | 2.06 (1.36-3.17) | **0.019*** |
| **CRP*, mg/L** | 0.98 (0.33-3.56) | 0.28 (0.22-0.42) | **0.041*** |
| ESR, mm/h | 11.5 (7.00-31.2) | 8.00 (5.75-11.2) | 0.141 |
| Ferritin, ng/ml | 230 (87.7-415) | 105 (66.6-170) | 0.096 |
| **β2-MG*, ng/ml** | 3022 ± 823 | 2247 ± 667 | **0.019*** |
| **Hepatic and Renal Function** | |  |  |
| **Albumin*, g/L** | 33.0 ± 5.95 | 38.9 ± 3.99 | **0.002**** |
| **Albumin to Globulin*** | 1.20 ± 0.30 | 1.47 ± 0.30 | **0.008**** |
| **Prealbumin*, mg/L** | 158 ± 47.1 | 226 ± 47.1 | **0.002**** |
| **Total protein*, g/L** | 61.3 ± 7.25 | 66.2 ± 6.24 | **0.041*** |
| CH50, U/ml | 39.4 (35.5-42.5) | 39.0 (29.0-41.0) | 0.797 |
| γ-GT, IU/L | 18.5 (13.0-28.2) | 20.0 (15.0-32.0) | 0.717 |
| ALT, IU/L | 30.5 (21.0-50.2) | 20.0 (13.0-30.0) | 0.088 |
| Alkaline phosphatase, IU/L | 58.5 (51.0-78.5) | 64.0 (51.0-74.0) | 0.918 |
| Direct bilirubin, μmol/L | 1.95 (1.67-2.52) | 1.90 (1.60-2.40) | 0.805 |
| Total bilirubin, μmol/L | 10.9 (9.65-13.5) | 11.1 (10.0-12.6) | 0.876 |
| Estimation of glomerular filtration rate | 103 (88.3-111) | 108 (90.4-115) | 0.707 |
| Creatinine, μmol/L | 61.0 (52.0-72.2) | 59.0 (56.0-68.0) | 0.958 |
| Uric acid, μmol/L | 280 (216-354) | 274 (251-325) | 0.848 |
| Glucose, mmol/L | 4.74 (4.31-5.20) | 5.17 (4.66-5.50) | 0.203 |
| **Blood Lipids** |  |  |  |
| **Total cholesterol*, mmol/L** | 3.92 ± 0.95 | 4.73 ± 1.06 | **0.028*** |
| Bile acid, μmol/L | 4.65 (2.98-6.47) | 5.20 (2.70-6.70) | 0.839 |
| LDL, mmol/L | 2.38 ± 0.79 | 2.83 ± 0.67 | 0.096 |
| Triglycerides, mmol/L | 1.48 (1.21-1.81) | 1.52 (1.26-2.29) | 0.754 |
| HDL, mmol/L | 0.94 ± 0.33) | 1.13 ± 0.28 | 0.080 |
| Free fatty acids, mmol/L | 0.45 (0.35-0.57) | 0.46 (0.32-0.48) | 0.548 |
| **ApoAI*, g/L** | 1.01 ± 0.27) | 1.25 ± 0.19 | **0.002**** |
| ApoB, g/L | 0.82 ± 0.22) | 0.86 ± 0.20 | 0.743 |
| ApoE, mg/dL | 4.35 (3.68-5.03) | 4.30 (3.50-5.75) | 0.918 |
| Lipoprotein(a), g/L | 0.10 (0.06-0.21) | 0.18 (0.11-0.21) | 0.224 |
| **Electrolytes** |  |  |  |
| Carbon dioxide, mmol/L | 27.1 ± 3.14 | 27.0 ± 1.95 | 0.918 |
| **Calcium*, mmol/L** | 2.07 ± 0.15 | 2.22 ± 0.12 | **0.002**** |
| Potassium, mmol/L | 3.85 ± 0.35 | 3.94 ± 0.29 | 0.524 |
| Phosphorus, mmol/L | 1.17 ± 0.16 | 1.24 ± 0.21 | 0.466 |
| Chlorine, mmol/L | 104 (102-106) | 104 (103-107) | 0.557 |
| Sodium, mmol/L | 141 (139-142) | 141 (139-142) | 0.761 |
| Magnesium, mmol/L | 0.83 (0.78-0.85) | 0.86 (0.81-0.89) | 0.208 |
| **Immunological indices** |  |  |  |
| IgG, g/L | 1330 (830-1554) | 1215 (1105-1335) | 0.828 |
| IgA, g/L | 216 (104-256) | 186 (110-246) | 0.828 |
| IgM, g/L | 79.5 (41.8-117) | 89.0 (52.0-120) | 0.743 |
| IgE, IU/mL | 61.7 (14.2-118) | 72.5 (32.5-121) | 0.848 |
| C3, g/L | 72.5 (59.5-78.8) | 79.5 (66.8-86.2) | 0.208 |
| C4, g/L | 18.0 (13.5-21.8) | 21.0 (14.8-25.0) | 0.761 |
| ASO, kIU/L | 37.0 (26.0-67.0) | 52.5 (29.0-62.5) | 0.786 |
| dsDNA IgG | 43.4 (28.8-80.9) | 32.5 (28.2-67.7) | 0.707 |
| **Tumor markers** |  |  |  |
| CEA, ng/mL | 1.90 (1.11-2.83) | 1.30 (0.81-1.99) | 0.437 |
| AFP, ng/mL | 2.48 (1.97-3.33) | 2.39 (2.17-4.06) | 0.761 |
| SCCA, ng/mL | 0.90 (0.60-1.45) | 0.80 (0.60-1.10) | 0.602 |
| **NSE*, ng/mL** | 21.7 (15.6-32.6) | 14.5 (11.9-16.4) | **0.008**** |
| CA125, U/mL | 12.5 (10.6-15.8) | 11.9 (8.15-15.7) | 0.743 |
| **CA153*, U/mL** | 13.2 (9.70-20.0) | 9.30 (6.38-10.9) | **0.015**** |
| CA199, U/mL | 4.95 (3.60-10.7) | 5.00 (3.20-10.7) | 0.862 |
| CA724, U/mL | 1.22 (0.86-2.38) | 1.03 (0.92-3.18) | 0.848 |
| **Coagulation markers** |  |  |  |
| APTT, s | 31.5 ± 3.12 | 30.2 ± 2.82 | 0.314 |
| Fg, g/L | 2.80 (2.50-3.50) | 2.40 (2.20-3.10) | 0.129 |
| INR | 0.99 (0.94-1.04) | 0.92 (0.86-1.00) | 0.110 |
| PT, s | 11.7 (11.1-12.2) | 10.9 (10.2-11.9) | 0.152 |
| TT, s | 18.0 (17.1-19.0) | 18.7 (18.4-19.3) | 0.129 |
| **Other comorbidities** |  |  |  |
| Hypertension | 6 (18.8%) | 7 (28.0%) | 0.409 |
| Diabetes mellitus | 4 (12.5%) | 3 (12.0%) | 0.954 |


1. *, *P* < 0.05; **, *P* < 0.01; ***, *P* < 0.001. P-values were adjusted using Benjamin-Hochberg false discovery rate (FDR).

2. Abbreviations: BMI, body mass index; CADM, clinically amyopathic dermatomyositis; CDM, classic dermatomyositis; ILD, interstitial lung disease; CIP, chronic interstitial pneumonia; A/SIP, acute/subacute interstitial pneumonia; TIF1-γ, transcription intermediary factor 1-gamma; MDA5, melanoma differentiation-Associated protein 5; ANA, anti-nuclear antibody; ENA, anti-extractable nuclear antigen antibody; LDH, lactate dehydrogenase; AST, aspartate aminotransferase; CK-MK, creatine kinase isoenzyme-MB; WBC, white blood cell count; RBC, red blood cell count; PLT, platelet count; Hb, hemoglobin; HCT, hematocrit; NLR, neutrophil/ lymphocyte ratio; CRP, C-reactive protein; ESR, erythrocyte sedimentation rate; β2-MG, beta-2-microglobulin; γ-GT, gamma-glutamyltransferase; ALT, alanine aminotransferase; LDL, low-density lipoprotein; HDL, high-density lipoprotein; Apo, apolipoprotein; ASO, anti-streptolysin O; dsDNA, double-stranded DNA; CEA, carcinoembryonic antigen; AFP, alpha-fetoprotein; SCCA, squamous cell carcinoma antigen; NSE, neuron specific enolase; CA, carbohydrate antigen; APTT, activated partial thromboplastin time; Fg, fibrinogen; INR, international normalized ratio; PT, prothrombin time; TT, thrombin time.
